# A Japanese boy with *NAA10*-related syndrome and hypertrophic cardiomyopathy

**DOI:** 10.1038/s41439-020-00110-0

**Published:** 2020-08-17

**Authors:** Ayumi Shishido, Naoya Morisada, Kenta Tominaga, Hiroyasu Uemura, Akiko Haruna, Hiroaki Hanafusa, Kandai Nozu, Kazumoto Iijima

**Affiliations:** 1 Department of General Medicine, Hyogo Prefectural Kobe Children’s Hospital, Kobe, Hyogo Japan; 2 Department of Pediatric Cardiology, National Cerebral and Cardiovascular Center, Suita, Osaka Japan; 3 Department of Clinical Genetics, Hyogo Prefectural Kobe Children’s Hospital, Kobe, Hyogo Japan; 4 Department of Pediatrics, Kobe University Graduate School of Medicine, Kobe, Hyogo Japan; 5 Department of Cardiology, Hyogo Prefectural Kobe Children’s Hospital, Kobe, Hyogo Japan; 6 Department of Pediatrics, Himeji Red Cross Hospital, Himeji, Hyogo Japan; 7 Department of Urology, Hyogo Prefectural Kobe Children’s Hospital, Kobe, Hyogo Japan; 8 Center for Medical Genetics, Shinshu University Hospital, Matsumoto, Nagano Japan

**Keywords:** Cardiac hypertrophy, Paediatric neurological disorders

## Abstract

*NAA10*-related syndrome is an extremely rare X-chromosomal disorder, the symptoms of which include intellectual disability (ID), ocular anomalies, or congenital heart diseases, such as hypertrophic cardiomyopathy (HCM). Here, we describe a 4-year-old Japanese male patient who exhibited mild ID, HCM, and specific facial features. A hemizygous mutation (NM_003491.3: c.455_458del, p. Thr152Argfs*6) in exon 7 of *NAA10* was detected. We recommend that patients undergo precise medical follow-up considering the characteristics of *NAA10*-related syndrome.

N-alpha-acetylation (N-terminal acetylation, NTA) is one of the most common protein modifications in eukaryotes, and ~80% of the N-termini of human proteins are acetylated^1^. *NAA10* (Xq28) encodes the enzyme N-alpha-acetyltransferase 10 (NAA10), which is the catalytic subunit of the N-terminal acetyltransferase A complex with the protein encoded by *NAA15* (4q31.1)^2^. NTA is essential for the preservation of normal cell function. However, its physiological significance has not been completely elucidated.

*NAA10* abnormality in humans was originally known as causing Ogden syndrome (MIM #300855), which is an X-chromosomal inherited disorder. Female patients with Ogden syndrome show mild to severe intellectual disability (ID), and male patients die early in life^3^. Recently, several male patients with various *NAA10* mutations have been reported to survive. These patients exhibit various phenotypes, such as hypertrophic cardiomyopathy (HCM)^4^, microphthalmia/anophthalmia^5^, or severe nonsyndromic ID^6^. These diseases due to *NAA10* abnormalities are collectively called *NAA10*-related syndrome^1^. We report a Japanese boy carrying a hemizygous *NAA10* mutation with HCM and ID but no microphthalmia.

Our patient was a Japanese boy who was the first child of healthy parents. He was delivered by elective cesarean section at 37 weeks of gestation because of a transverse position, and his Apgar score was 8/8 (at 1 and 5 min). His birth weight was 2170 g (small for gestational age). He exhibited genital abnormalities (split scrotum, hypospadias), eyelid drooping, and bilateral overlap of toes at birth. He was diagnosed with congenital heart disease (CHD) by echocardiography. His karyotype was 46,XY.

At 1 month of age, he was referred to our hospital for examination for CHD. Echocardiogram revealed a perimembranous outlet ventricular septal defect (defect: 5.0 mm × 4.5 mm), an atrial septal defect (defect: 3.5 mm × 3.6 mm), and left ventricle (LV) wall thickness (interventricular septum: 5.4 mm; left ventricular posterior wall: 3.7 mm) (Fig. 1a). At the age of 5 months, echocardiogram showed thickening of the LV, especially the interventricular septum. Thus, he was diagnosed with HCM with LV outflow tract obstruction (LVOTO) and trivial-mild mitral regurgitation (MR). After 1 month, the MR and LVOTO worsened, and β-blocker therapy was started.

**Fig. 1:**
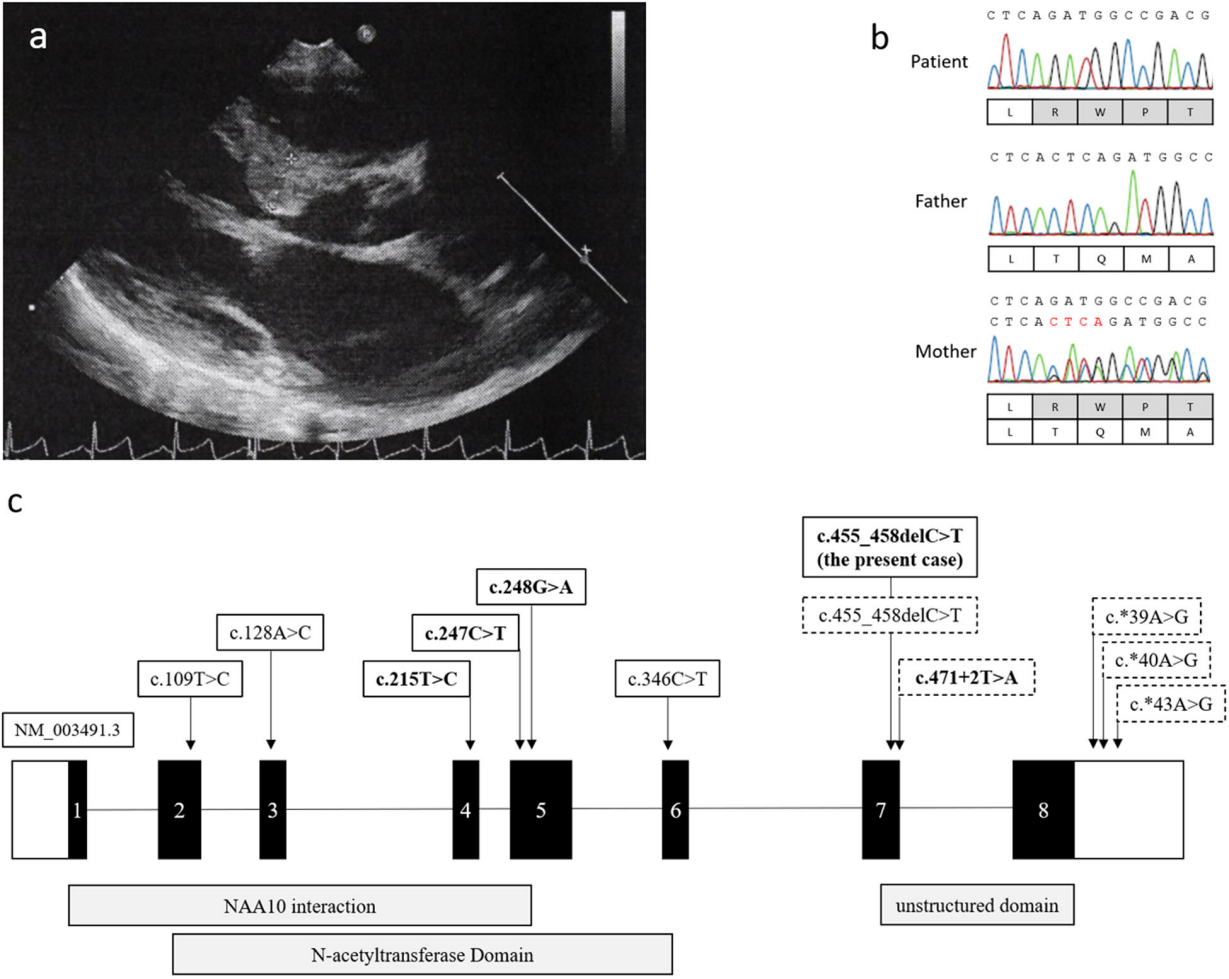
The results of the cardiac and genetic testing in the patient. **a** Echocardiography of the patient at 1 year of age. **b** *NAA10* mutations were identified in the patient and his mother by the Sanger method. Red letters indicate deleted nucleotides. **c** The genotype of the *NAA10* mutation in male patients of the present case and literature. The bold face indicates hypertrophic myopathy, and the dotted line shows microphthalmia or anophthalmia.

At the age of 1 year, he was referred to the Clinical Genetics Department for molecular diagnosis. He showed mild ID, characteristic facial features, including eyelid drooping, exophthalmos, underdeveloped superior crus of antihelix, unilateral hearing loss, bifid scrotum, hypospadias, perodactylia, and CHD. His height at the age of 1 year was 69.6 cm (−2.0 SD), and his body weight was 8.4 kg (body mass index (BMI) 17.3). Routine blood tests and serum amino acid analysis were normal. He started to walk independently at the age of 1 year and 7 months. At the age of 4 years, his height was 90.0 cm (−2.63 SD), and his body weight was 12.2 kg (BMI 15.1). He was able to trot around, but he was unable to speak meaningful words. Brain magnetic resonance at the age of 1 year and 6 months imaging did not show any abnormalities.

To confirm his molecular diagnosis, we analyzed DNA samples derived from his peripheral blood by next-generation sequencing using TruSight One (Illumina, San Diego, CA, USA) after obtaining written informed consent from his parents. All procedures were reviewed and approved by the Institutional Review Board of Kobe University School of Medicine (86) and Hyogo Prefectural Kobe Children’s Hospital (28-4) and were in accordance with the ethical standards of the Declaration of Helsinki. We identified a hemizygous four-base deletion (NM_003491.3: c.455_458del, p. Thr152Argfs*6) in exon 7 of *NAA10*. The same deletion was identified in his asymptomatic mother, although in the heterozygous state (Fig. 1b). Other pathogenic variants, including genes associated with RASopathies, were not identified by TruSight One.

Male patients with *NAA10*-related syndrome are extremely rare and present various clinical features. Previously, it was considered that boys with *NAA10* mutations die early in life^2^. To our knowledge, 35 male patients from 13 families with 11 types of genetic aberrations in *NAA10* have been reported (Table 1). The frequently observed symptoms are as follows: ID, motor developmental delay, growth failure, ophthalmic diseases, skeletal disorders, including scoliosis or digital anomalies, and cardiac disorders. Most *NAA10* mutation types in male patients are missense variants, but three families, including the present case, harbor truncated mutations^2,7^. Patients with *NAA10* mutations in further unstructured domains (exons 7 or 8) tend to exhibit microphthalmia or anophthalmia (Fig. 1c). The mutation of the present patient is a frameshift mutation that is identical to that of a boy reported by Cheng^3^. We presumed that he survived because the mutation is in a region where nonsense-mediated degradation does not occur. However, our patient did not show microphthalmia/anophthalmia. Further investigations are needed to clarify the precise mechanism of microphthalmia in *NAA10*-related syndrome. Cardiac complications are also observed in male patients with *NAA10*-related syndrome. However, the genotype–phenotype correlation is not clear. In addition, the exact cause of HCM in the present patient remains unknown.

**Table 1:** Phenotypes and genotypes of male patients with *NAA10*-related syndrome.

Author	Rope et al.^3^	Casey et al.^8^	Støve et al.^4^	Saunier et al.^9^	Ree et al.^10^	Popp et al.^6^	Esmailpour et al.^5^	Cheng et al.^2^	Slavotinek and Lee^11^, Johnston et al.^7^	Johnston et al.^7^	Johnston et al.^7^	The present case
Gender	Male	Male	Male	Male	Male	Male	Male	Male	Male	Male	Male	Male
Variant	c.109 T > C	c.128A > C	c.215T > C	c.247C > T	c.248G > A	c.346C> T	c.471 + 2T > A	c.455_458del	c.*39A > G	c.*40A > G	c.*43A > G	c.455_458del
Amino acid	p.Ser37Pro	p.Tyr43Ser	p.Ile72Thr	p.Arg83Cys	p.Arg83His	p.Arg107Phe	p.Glu157fs*45	p.Thr152Argfs*6				p.Thr152Argfs*6
Inheritance	Maternal	Maternal	Maternal	Maternal	Maternal	De novo	Maternal	Maternal	Maternal	Maternal	Maternal	Maternal
Number of patients	8 (2 families)	2 (1 family)	3 (2 families)	1	2 (1 family)	1	4 (1 family)	1	5 (1 family)	1	7 (1 family)	1
Age at last investigation	5–16 m	20–25 y	3–8 y	NA	12–15 y	5 y	NA	11 y	NA	8 m	NA	4 y
Birth weight (kg)	1.5–3.3	NA (normal)	3.6–3.8	3.2	3.2–3.3	NA (normal)	NA	NA	NA	NA	NA	2.1
Growth failure	+	+	−	NA	+	+	+	+	NA	−	NA	+
Neurological	Cerebral atrophy, hypotonia	Dilation of LV, hypotonia, seizures,	Medulloblastoma, mild PVL, relative paucity of frontal lobe, thin CC,	Hypotonia	Hypotonia, seizures	Hypotonia	ASCVD, seizure	hypotonia	NA	Chiari II malformation, hydrocephalus, myelomeningocele, spina bifida	Neural tube defect	Hypotonia
Intellectual disability	+	+	+	NA	+	+	+	+	−	−	−	+
Motor delay	+	NA	+	NA	+	+		+	NA	−	NA	+
Cardiac disorder	Arrhythmia, PAS, PDA, VSD	LQT, VT	HCM	HCM, PH, SVT	HCM	−	r VH	ASD	NA	−	NA	HCM, ASD, VSD
Ocular disorder	Prominent eyes	r Amblyopia, astigmatism, r convergent squint	−	NA	Astigmatism	−	b Anophthalmia, microphthalmia	Microcornea, microphthalmia	u Anophthalmia,	u Phthisis bulbi	r Anophthalmia	Astigmatism, esotropia
Facial feature	Flared nares, large ears, narrow palate	Downslanting palpebral fissures	High arched palate, rather thick lips, wide spaced teeth,	NA	Closely spaced eyes, tented upper lip	Deep set eyes, diastema, large ears, long eyelashes, prominent forehead	High arched palate, large abnormally formed ears	−	NA	Downturned corners of the mouth, widely spaced eyes	NA	Eyelid drooping, external ear anomaly
Skeletal disease	Broad or widely spaced toes, clinodactyly, delayed osseous development, large fontanels, metatarsal valgus, scoliosis	b Acetabular dysplasia, b valgus deformity, scoliosis, toe syndactyly	Barrel chest, delayed closure of fontanelle	Hallux varus, sandal gap	−	−	Pectus excavatum, pes planus, scoliosis, toe syndactyly	Clubfeet, pectus excavatum, scoliosis, syndactyly	Six toes	Small feet with upturned nail	b 2–3 cutaneous syndactyly of toe	b Overlaps of toes
Kidney and urinary system	Cryptorchidism	−	−	Small cortical cysts	−	Hypoplastic scrotum		Hypospadias	NA	Small penis, l VUR		Bifid scrotum, hypospadias
Others	Inguinal hernia, died at <2 years	Congenital pneumonia, distended veins, inguinal hernia	Inguinal hernia	NA	Chronic constipation, sparse scalp hair	−		Agenesis of CC, craniosynostosis				Fair skin, l hearing loss

*ASCVD* arteriosclerotic vascular disease, *ASD* atrial septal defect, *b* bilateral, *CC* corpus callosum, *HCM* hypertrophic cardiomyopathy, *l* left, *LQT* long QT interval, *LV* lateral ventricles, *m* months, *NA* not available, *PAS* pulmonary artery stenosis, *PDA* persistent ductus arteriosus, *PVL* periventricular leukomalacia, *r* right, *SVT* supraventricular tachyarrhythmia, *u* unilateral, *VH* ventricular hypertrophy, *VSD* ventricular septal defect, *VT* ventricular tachycardia, *VUR* vesicoureteral reflux, *y* years.

Female patients with *NAA10*-related syndrome may display various types of ID^6^. The mother of the present patient carried an identical *NAA10* mutation in the heterozygous state, but she did not exhibit any medical abnormalities. Families with *NAA10*-related syndrome should be considered during genetic counseling with regard to recurrence in the next child, irrespective of the sex of the child.

In conclusion, we report for the first time a Japanese male patient with *NAA10*-related syndrome. We recommend that patients undergo precise medical follow-up, particularly for neurodevelopment, cardiac disease, including HCM, ocular abnormalities, and scoliosis. The results of our study are useful for the recognition of *NAA10*-related syndrome.

## Data Availability

The relevant data from this Data Report are hosted at the Human Genome Variation Database at 10.6084/m9.figshare.hgv.2879.
